# Endocytic deficiency induced by ITSN-1s knockdown alters the Smad2/3-Erk1/2 signaling balance downstream of Alk5

**DOI:** 10.1242/jcs.163030

**Published:** 2015-04-15

**Authors:** Cristina Bardita, Dan N. Predescu, Fei Sha, Monal Patel, Ganesh Balaji, Sanda A. Predescu

**Affiliations:** 1 Department of Pharmacology, Rush University, Chicago, IL 60612, USA; 2 Pulmonary and Critical Care Medicine, Rush University Medical Center, Chicago, IL 60612, USA; 3 Department of Microbiology and Immunology, College of Medicine, University of Illinois at Chicago, Chicago, IL 60612, USA

**Keywords:** Microparticle, Alternative endocytic pathway, Proliferation

## Abstract

Recently, we demonstrated in cultured endothelial cells and *in vivo* that deficiency of an isoform of intersectin-1, ITSN-1s, impairs caveolae and clathrin-mediated endocytosis and functionally upregulates compensatory pathways and their morphological carriers (i.e. enlarged endocytic structures, membranous rings or tubules) that are normally underrepresented. We now show that these endocytic structures internalize the broadly expressed transforming growth factor β receptor I (TGFβ-RI or TGFBR1), also known as Alk5, leading to its ubiquitylation and degradation. Moreover, the apoptotic or activated vascular cells of the ITSN-1s-knockdown mice release Alk5-bearing microparticles to the systemic circulation. These interact with and transfer Alk5 to endocytosis-deficient endothelial cells, resulting in lung endothelial cell survival and phenotypic alteration towards proliferation through activation of Erk1 and Erk2 (also known as MAPK3 and MAPK1, respectively). We also show that non-productive assembly of the Alk5–Smad–SARA (Smad anchor for receptor activation, also known as ZFYVE9) signaling complex and preferential formation of the Alk5–mSos–Grb2 complex account for Erk1/2 activation downstream of Alk5 and proliferation of pulmonary endothelial cells. Taken together, our studies demonstrate a functional relationship between the intercellular transfer of Alk5 by microparticles and endothelial cell survival and proliferation, and define a novel molecular mechanism for TGFβ and Alk5-dependent Erk1/2^MAPK^ signaling that is significant for proliferative signaling and abnormal growth.

## INTRODUCTION

Acute lung injury (ALI) or mild acute respiratory distress syndrome (ARDS), according to the Berlin definition (Ranieri et al., 2012), are associated with excessive apoptosis of endothelial and epithelial cells (Henson and Tuder, 2008; Le et al., 2008; Predescu et al., 2013). Although apoptosis might induce pulmonary endothelial and epithelial barrier dysfunction leading to pulmonary edema, evidence suggests that apoptosis plays a beneficial role during ALI resolution owing to the pro-regenerative role of clearance of apoptotic cells (Predescu et al., 2013; Schmidt and Tuder, 2010). This effect is mediated through the production of growth factors including TGFβ by macrophages engulfing apoptotic cells, or perhaps by other vascular cells (Bardita et al., 2013; Henson and Tuder, 2008). TGFβ, owing to its anti-inflammatory properties confines the extent of septal injury and speeds recovery in ALI (D'Alessio et al., 2009).

We have recently shown that *in vivo* deficiency of ITSN-1s, an isoform of ITSN-1 that is highly prevalent in lung endothelium and deficiency of which is relevant to the pathology of ALI/ARDS (Bardita et al., 2013; Predescu et al., 2013), induces extensive lung endothelial cell apoptosis and injury; after only 7 days of ITSN knockdown (KD-ITSN), the remaining endothelial cells exhibited phenotypic changes including hyperproliferation and apoptosis resistance against ITSN-1s deficiency, leading to increased microvessel density, repair and remodeling of the injured lung. Under pathological conditions, dysfunctional endothelial cells also show altered intracellular trafficking and signaling of cell surface receptors, such as TGFβ-RI, which is implicated in the pathogenesis of ALI/ARDS (Kranenburg et al., 2002; Morrell et al., 2001; Sehgal and Mukhopadhyay, 2007; Voelkel and Cool, 2003). Endocytic dysfunction and non-productive assembly of the endocytic machinery might alter canonical signaling pathways with detrimental consequences for endothelial cell function (Mukherjee et al., 2006; Sorkin and von Zastrow, 2009). Although endothelial cells alone are insufficient to cause ALI (Wiener-Kronish et al., 1991), their injury or dysfunction and activation, as well as their interaction with the alveolar epithelium are crucial not only for the onset of ALI/ARDS, but also for repair and remodeling of the injured lung.

Emerging *in vivo* and *in vitro* evidence has revealed a crucial role of circulatory microparticles as transcellular delivery systems and in the communication between different cell types; microparticles are present in healthy and pathological settings; they store important bio-effectors and induce endothelial modifications, angiogenesis or differentiation (Mause and Weber, 2010). Although the presence of microparticles in ALI/ARDS has been reported (McVey et al., 2012), their *in vivo* relevance in the modulation of signaling pathways leading to improved endothelial and vascular functions in the setting of lung injury has not been explored. Given that ITSN-1s deficiency in cultured endothelial cells triggers mitochondrial apoptosis (Predescu et al., 2007a), whereas, *in vivo*, it leads to the emergence of proliferative and apoptosis-resistant endothelial cells (Bardita et al., 2013), we hypothesized that the *in vivo* microparticles released by apoptotic or activated vascular cells in the systemic circulation of KD-ITSN mice might account for endothelial cell survival and alterations in their phenotype. We now demonstrate a functional relationship between the intercellular transfer of Alk5 by microparticles and endothelial cell survival and proliferation, and define a novel molecular mechanism for TGFβ–Alk5-dependent Erk1 and Erk2 (also known as MAPK3 and MAPK1, respectively; hereafter referred to as Erk1/2^MAPK^) signaling, significant for the abnormal proliferation of pulmonary endothelial cells.

## RESULTS

### Endocytic deficiency caused by KD-ITSN modifies Alk5 endocytic trafficking and enhances its degradation

**Fig. 1.**
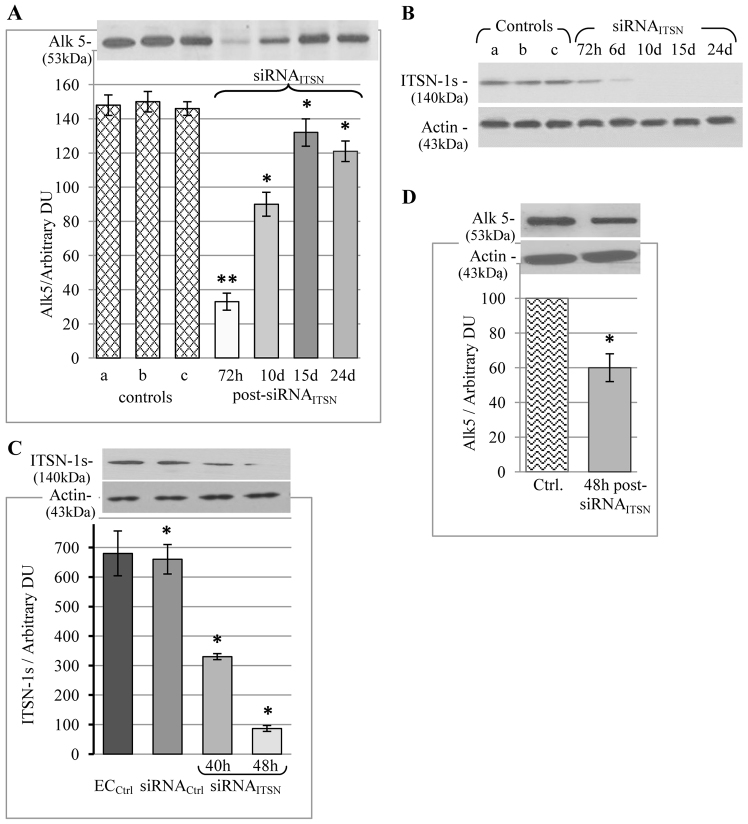
**KD-ITSN mouse lungs and EC_KD-ITSN_ show decreased Alk5 protein expression.KD-ITSN mouse lungs and EC_KD-ITSN_ show decreased Alk5 protein expression.** (A) Western blot of Alk5 from lung lysates (70 µg total protein) of wild-type mice (a), empty-liposome- (b) and siRNA_Ctrl_-treated (c) mice, as well as lung lysates from KD-ITSN mice at different time points post siRNA treatment, as indicated. Values are shown as the mean±s.e.m. *P<0.01; **P<0.05 versus controls. (B) Lung lysates (70 µg/lane) of the same controls as shown in panel A and siRNA_ITSN_-treated mice were analyzed by western blotting with antibodies against ITSN-1 and actin (as the loading control), at several time points post delivery; *n* = 3 mice for each experimental condition and time point considered. (C) Cell lysates (50 µg of total protein) of non-transfected cells (EC_Ctrl_), endothelial cells transfected with the siRNA_Ctrl_ and endothelial cells transfected with siRNA_ITSN_ were analyzed by SDS-PAGE and immunoblotting with anti-ITSN primary antibody. Actin served as the loading control. Values are shown as the mean±s.e.m. *P<0.01 versus EC_Ctrl_. (D) Cultured EC_Ctrl_ and EC_KD-ITSN_, 38–40 h post siRNA_ITSN_ transfection, were subjected to SDS-PAGE, electrotransfer to nitrocellulose and western blotting for Alk5 protein expression; actin served as the loading control. DU, densitometric units. Values are shown as the mean±s.e.m. *P<0.01 versus EC_Ctrl_. All data are representative of least three independent experiments.

Recently, we investigated the *in vivo* effects of long-term ITSN-1s deficiency on pulmonary vasculature and lung homeostasis, using a KD-ITSN mouse model generated by repeated delivery of a specific small interfering (si)RNA targeting ITSN-1 (siRNA_ITSN_; Bardita et al., 2013; Predescu et al., 2012). We have shown that acute ITSN-1s deficiency in the murine lungs results in a significant decrease in Erk1/2^MAPK^ pro-survival signaling, increased endothelial cell apoptosis and lung injury; at 24 days post siRNA_ITSN_ initiation, the surviving endothelial cells showed reactivation of Erk1/2^MAPK^ and phenotypic changes towards proliferation. The threefold increase in mature TGFβ expression at 10 days post siRNA_ITSN_ treatment compared with that of control mice suggested that TGFβ signaling might account for Erk1/2^MAPK^ activation in KD-ITSN mice. Because TGFβ elicits its signaling by binding to its cell surface Ser/Thr kinase receptors, leading to the formation of heterocomplexes between Alk5 (also known as TGFBR1) and transforming growth factor β type II receptor (TGFβ-RII or TGFBR2) (Lebrin et al., 2005), and because Alk5 expression might play an important regulatory role in TGFβ signaling, we performed a timecourse analysis of Alk5 protein expression in the lung lysates of the KD-ITSN mice. At 72 h post siRNA_ITSN_ delivery, Alk5 expression was 80% lower, compared with that of all controls [wild type (wt), empty-liposome-treated and si*CONTROL* non-targeting siRNA (siRNA_Ctrl_)-treated mice; Fig. 1A]. Later on, at 10 days, 15 days and 24 days post siRNA_ITSN_ delivery, Alk5 expression showed a gradual increase, reaching values relatively close to those of controls. The expression of ITSN-1s protein was monitored at several time points after siRNA_ITSN_ delivery by using western blotting of mouse lung lysates (Fig. 1B); at day 3, it was ∼75% lower relative to expression in all control mice, and the knockdown was maintained for the next 21 days. Delivery of empty liposomes (Fig. 1B, lane b) or liposomes containing the non-specific siRNA (Fig. 1B, lane c), did not affect the level of ITSN-1s protein, compared with that of untreated mice (Fig. 1B, lane a). Actin served as loading control, Fig. 1B (lower panel). Comparable ITSN-1s downregulation, with the same timeline as in the lung, was detected in the brain, although knockdown in the heart, kidneys and liver was less efficient (Bardita et al., 2013).

Because ITSN-1s deficiency functionally upregulates alternative transport pathways and their carriers to compensate for impaired endocytosis mediated by caveolae and clathrin-coated vesicles (Predescu et al., 2012) involved in Alk5 intracellular trafficking (Derynck and Zhang, 2003), we also investigated Alk5 expression and internalization in cultured endothelial cells deficient for ITSN-1s (EC_KD-ITSN_). The ITSN-1 gene was specifically and efficiently knocked down using an siRNA approach that has been described previously (Predescu et al., 2007a). EC_KD-ITSN_ were used at 38–40 h post siRNA_ITSN_ transfection, a time point when the protein expression is 50% lower compared with that of controls (Fig. 1C) and endothelial cells are not yet apoptotic (not shown), as determined by TUNEL as described previously (Predescu et al., 2007a). Actin served as the loading control (Fig. 1C). The transfection of endothelial cells with si*CONTROL* non-targeting siRNA did not affect the expression of ITSN-1s at 40 h post transfection. The expression of Alk5 protein in EC_KD-ITSN_ was 40% of the levels observed in controls, as indicated by western blotting with an anti-Alk5 antibody followed by densitometry; actin was used as the loading control (Fig. 1D).

**Fig. 2.**
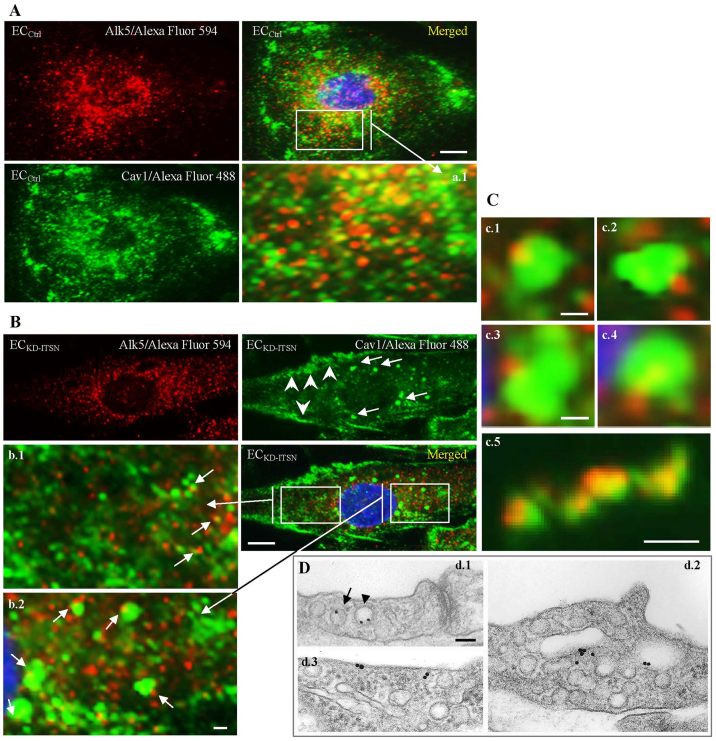
**Endocytic deficiency caused by KD-ITSN alters Alk5 endocytic trafficking.Endocytic deficiency caused by KD-ITSN alters Alk5 endocytic trafficking.** (A,B) Alk5 (Alexa Fluor 594) and cav1 (Alexa Fluor 488) double immunofluorescence of EC_Ctrl_ revealed strong, fine puncta for both cav1 and Alk5; in EC_KD-ITSN_ (B, arrowheads), cav1 accumulates at the cell periphery. The merged images reveal significant colocalization of Alk5 and cav1 in EC_Ctrl_ (A,a1); however Alk5 and cav1 colocalization is more limited in EC_KD-ITSN_ (B, merged image and inset b.1, arrows). Large fluorescent puncta immunoreactive to both cav1- and Alk5-specific antibodies (inset b.2, arrows) were detected inside the cell. (C,c.1–c.4) Gallery of magnified Alk5 and cav1 double-positive structures present in EC_KD-ITSN_; highly magnified cav1 and Alk5-positive puncta in EC_Ctrl_ (c5) are shown for comparison. (D) Pre-embedding EM immunocytochemistry demonstrates the association of 8-nm gold particles conjugated to the Alk5 antibody with caveolae (d1, arrow) and CCVs (d1, arrowhead) in wild-type mice. In KD-ITSN specimens, gold particles label the endothelial plasma membrane (d3), as well as the enlarged endocytic or tubulovesicular structures (d2). Scale bars: 10 µm (A,B), 5 µm (b.1,b.2), 0.6 µm (c.1), 0.75 µm (c.2), 1.0 µm (c.3), 0.8 µm (c.4), 0.9 µm (c5), 100 nm (D). All data are representative of least three independent experiments.

Next, untreated endothelial cells (EC_Ctrl_) and EC_KD-ITSN_ were subjected to immunofluorescent staining for Alk5 and caveolin-1 (cav1). Anti-Alk5 antibody followed by an Alexa-Fluor-594-conjugated secondary antibody revealed a strong punctate pattern throughout the cytoplasm in both EC_Ctrl_ (Fig. 2A) and EC_KD-ITSN_ (Fig. 2B). When antibody against cav1 followed by Alexa-Fluor-488-conjugated secondary antibody was used, both EC_Ctrl_ (Fig. 2A) and EC_KD-ITSN_ (Fig. 2B) displayed small fluorescent puncta, most likely caveolae. Cav1/Alexa Fluor 488 staining of EC_KD-ITSN_ also revealed large fluorescent structures (Fig. 2B, arrows), possibly the counterparts of the large tubulovesicular structures detected by electron microscopy (EM) in EC_KD-ITSN_ (Bardita et al., 2013; Predescu et al., 2012). In addition, the increased cav1 immunoreactivity at the cell periphery in EC_KD-ITSN_ (Fig. 2B, arrowheads), was consistent with impaired caveolae internalization. When the colocalization of Alk5 and cav1 was analyzed, EC_Ctrl_ showed significant Alk5 colocalization with cav1 (Fig. 2A, merged image, inset a.1). However, colocalization of Alk5 and cav1 was limited in EC_KD-ITSN_ (Fig. 2B, merged image). Only few Alk5-positive puncta colocalized with cav1 (b.1, arrows). Significantly, Alk5 immunoreactivity colocalized with the large cav1-positive puncta (Fig. 2B,b.2; Fig. 2C,c.1–c.4), suggesting a possible involvement of these cav1-positive structures in Alk5 endocytic trafficking. Several cav1-positive puncta, most likely discrete caveolae, associated with Alk5 immunoreactivity are shown for comparison (Fig. 2C,c.5). Morphometric analyses of highly magnified large endocytic structures indicated that 70% are both cav1- and Alk5-positive and 24% were only cav1-positive. For the remaining 6%, it was difficult to conclude on cav1 and Alk5 colocalization.

In addition, pre-embedding immuno-EM for Alk5 (Fig. 2D), indicated that in the lung endothelial cells of KD-ITSN mice, 8-nm gold-conjugated Alk5 antibody labels the cell surface (d3) and is apparently internalized and associated with large endocytic tubulovesicular structures (d2). In wild-type mouse lung endothelial cells, Alk5 antibody labels caveolae and CCVs (d1), endothelial plasma membrane and occasionally endosomal structures (not shown). Taken together, the observations suggest that perturbation of caveolae-mediated endocytosis due to ITSN-1s deficiency upregulates cav1-dependent alternative endocytic pathways and their morphological structures or carriers, which are underrepresented under normal conditions (Doherty and McMahon, 2009; Predescu et al., 2012), and that these structures might be involved in Alk5 endocytic trafficking.

**Fig. 3.**
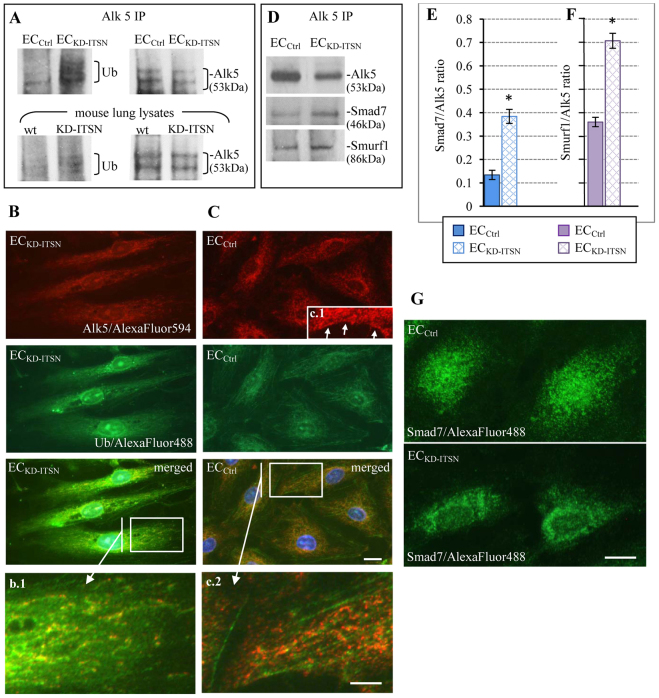
**Endocytic deficiency caused by KD-ITSN enhances degradation of Alk5.Endocytic deficiency caused by KD-ITSN enhances degradation of Alk5.** (A) Lysates of EC_Ctrl_, EC_KD-ITSN_, wild-type (wt) and KD-ITSN mice, normalized to 500 µg of total protein, were subjected to immunoprecipitation (IP) using the anti-Alk5 antibody. Western blotting using a ubiquitin (Ub)-specific antibody shows significant ubiquitylation of Alk5 in ITSN-deficient specimens. Anti-Alk5 pulls down Alk5 in controls and KD-ITSN samples; however, Alk5 amounts are ∼40% lower in KD-ITSN samples, as estimated by densitometry (NIH ImageJ). (B,C) Double immunofluorescence for Alk5 (Alexa Fluor 594) and ubiquitin (Alexa Fluor 488) in EC_KD-ITSN_ and EC_Ctrl_. Panels b.1 and c.1 are magnifications of boxed areas in the merged images from B and C, respectively. Arrows show the labeling of endothelial cell plasma membrane by Alk5 antibody. (D) Lysates of EC_Ctrl_ and EC_KD-ITSN_ were subjected to immunoprecipitation with an Alk5-specific antibody, followed by western blotting for Alk5, Smad7 and Smurf1. Note the decreased Alk5 immunoreactivity in EC_KD-ITSN_ compared with that of EC_Ctrl_. (E,F) Densitometry of Alk5, Smad7 and Smurf1 immunoreactivity. Values are shown as the mean±s.e.m. of the ratios Smad7:Alk5 (blue bars) and Smurf1:Alk5 (purple bars); *P<0.05 versus controls. (G) EC_Ctrl_ and EC_KD-ITSN_ subjected to Smad7 (Alexa Fluor 488) fluorescent staining. Scale bars: 20 µm (B,C,G); 10 µm (b.1, c.2). All data are representative of least three independent experiments.

Because caveolae internalization sends Alk5 to the ubiquitylation machinery (Itoh and ten Dijke, 2007), we next investigated whether the decreased expression of Alk5 might be caused by increased ubiquitylation. Alk5 immunoprecipitation followed by immunoblotting with an antibody against ubiquitin applied to EC_Ctrl_ and EC_KD-ITSN_ (Fig. 3A), as well as on lung lysates of wild-type and KD-ITSN mice, 10 days post siRNA_ITSN_ initiation (Fig. 3A), confirmed significant Alk5 ubiquitylation in ITSN-deficient specimens compared with that of controls. Moreover, double immunofluorescence for Alk5 and ubiquitin in EC_KD-ITSN_ revealed a punctate pattern of Alk5 in the cytosol (Fig. 3B), no significant plasmalemma staining and prominent colocalization with ubiquitin immunofluorescence (Fig. 3B, merged image). EC_Ctrl_ showed increased Alk5 immunoreactivity in the cytosol and at the plasma membrane compared with that of EC_KD-ITSN_ (Fig. 3C,c.1), some colocalization of Alk5 and ubiquitin and a significant pool of Alk5 not colocalizing with ubiquitin (Fig. 3C,c.2). The panels b.1 and c.2 show for comparison the magnified boxed areas in the merged images in Fig. 3B and Fig. 3C, respectively; although under control conditions, colocalization between Alk5 and ubiquitin is limited, in EC_KD-ITSN_, Alk5 and ubiquitin were significantly colocalized, consistent with Alk5 ubiquitylation and degradation; moreover, the decreased Alk5 immunoreactivity in EC_KD-ITSN_ compared with that of EC_Ctrl_ is consistent with decreased Alk5 expression in these ECs. In the ubiquitin–proteasome pathway, the HECT-type E3 ubiquitin ligases (Smurf1 and Smurf2) interact with the nuclear Smad7 (a negative regulator of TGFβ signaling) and induce its nuclear export, followed by assembly of the Smad7–Smurfs–Alk5 complex and enhanced turnover of Alk5 by ubiquitylation (Murakami et al., 2010). Alk5 immunoprecipitation followed by immunoblotting with antibodies against Smad7 and Smurf1 applied on endothelial cell lysates indicated an increased association of both Smad7 and Smurf1 with Alk5 in EC_KD-ITSN_ by comparison to EC_Ctrl_ (Fig. 3D). However, because Alk5 amounts are ∼40% lower in KD-ITSN samples, as estimated by densitometry, the ratios of Smad7:Alk5 (Fig. 3E) and Smurf1:Alk5 (Fig. 3F) are significantly higher in EC_KD-ITSN_ compared with EC_Ctrl_, consistent with increased Alk5 degradation. We also detected translocation of Smad7 from the nucleus to the cytosol in EC_KD-ITSN_, whereas the EC_Ctrl_ showed significant Smad7 nuclear immunoreactivity (Fig. 3G). Taken together, these observations demonstrate that ITSN-1s deficiency alters the endocytic trafficking of Alk5, causing its enhanced degradation.

### The apoptotic or activated circulating and vascular cells of KD-ITSN mice release elevated levels of microparticles comprising Alk5 into the bloodstream

**Fig. 4.**
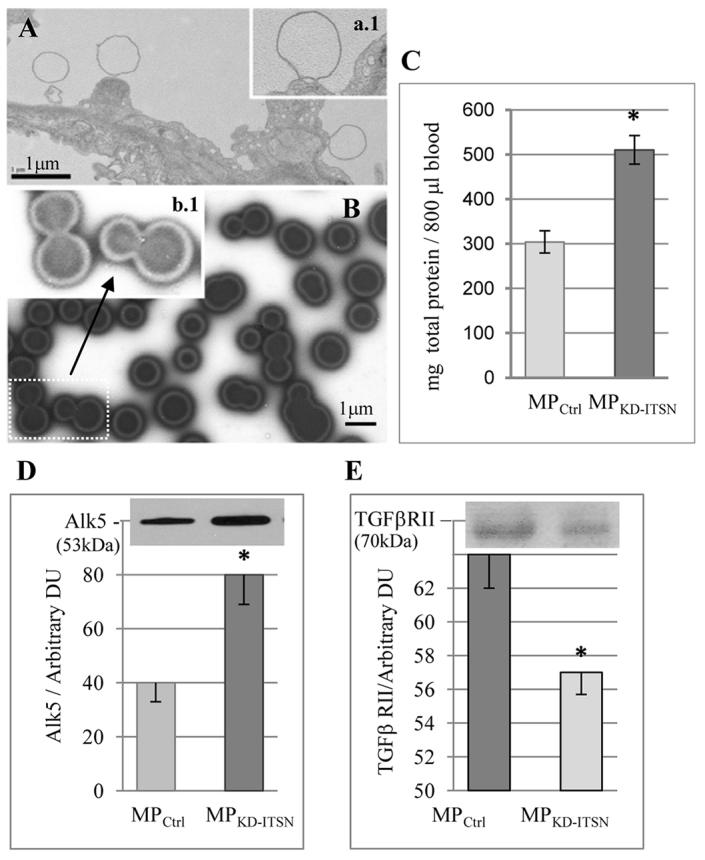
**The apoptotic or activated circulating and vascular cells of KD-ITSN mice release elevated levels of microparticles into the bloodstream.The apoptotic or activated circulating and vascular cells of KD-ITSN mice release elevated levels of microparticles into the bloodstream.** (A,a1) EM of a mouse lung endothelial cell (10 days post siRNA_ITSN_ delivery) shows small vesicular structures in the lumen of the blood vessel. (B) Negative staining EM of MP_KD-ITSN_ (10 days post siRNA_ITSN_ delivery) demonstrates their ability to fuse to each other (b1). (C) Increase in abundance of microparticles at 10 days post siRNA_ITSN_. Total protein amounts are normalized to 800 µl of blood and plotted as the means±s.e.m.; *n* = 3; *P<0.01. (D,E) Microparticles (15 µl volume; normalized to equivalent µl of blood) were analyzed for the expression of Alk5 and TGFβ-RII, respectively, as above. *n* = 3; *P<0.01. DU, densitometric units. Scale bars: 1 µm (A,B). All data are representative of three independent experiments.

**Fig. 5.**
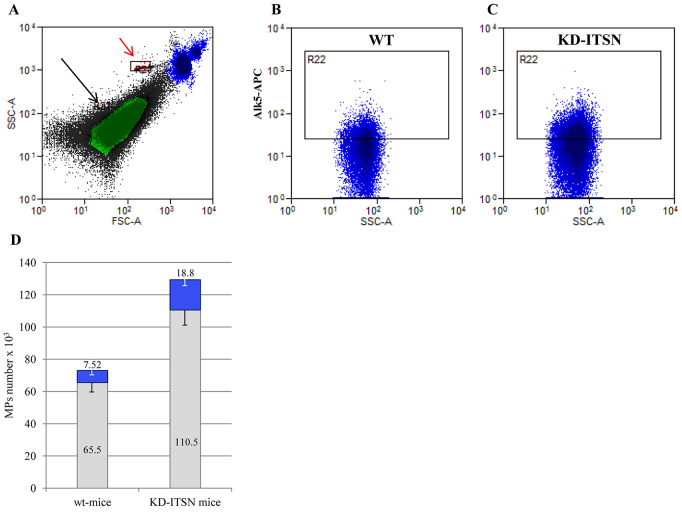
**Flow cytometry analyses of microparticles.Flow cytometry analyses of microparticles.** (A) The microparticle gate was determined using 1.35-µm Spherotech nano fluorescent size standard beads (a; black arrow). The red arrow in A shows the counting beads (3 µm diameter). (B,C) Representative plots of flow cytometry studies for identification of Alk5-positive microparticles. WT, wild type. (D) An increase in the total number of microparticles (MPs; blue and gray bars together) as well as an increase in Alk5-expressing microparticles (blue bars only) was observed in KD-ITSN mice compared with wild-type mice. Data show the mean±s.e.m.; *n* = 3 mice for each experimental condition; *P<0.01 versus wild-type mice. Data are representative of three independent experiments.

EM analyses of KD-ITSN mouse lungs revealed frequently in the lumen of the blood vessels the presence of microparticles with 0.5–1.0 µm diameter, many of them membrane-bound to endothelial cells (Fig. 4A,a1). Because microparticles might be a means to replenish endothelial cells with Alk5, we isolated the microparticles from the blood of KD-ITSN mice (MP_KD-ITSN_) at 10 days post siRNA_ITSN_ initiation and subjected them to negative-staining EM. MP_KD-ITSN_ are abundant, display double-membrane morphology and notably undergo membrane fusion and communicate with each other (Fig. 4B,b1). *In vivo* MP_KD-ITSN_ release, evaluated by quantification of the amount of the total protein in the isolated microparticles, indicated the highest amount, a ∼44% increase compared with controls (Fig. 4C), at day 10 post siRNA_ITSN_, when endothelial cell apoptosis was at its peak (Bardita et al., 2013). Next, equal volumes of MP_Ctrl_ and MP_KD-ITSN_ (normalized to equivalent µl of blood) were analyzed for their Alk5 content; Alk5 expression was significantly higher in MP_KD-ITSN_ (Fig. 4D), consistent with the idea that in the systemic circulation of the KD-ITSN mice there are more Alk5-positive microparticles compared with wild-type mice. MP_KD-ITSN_ were also immunoreactive to the vascular endothelial growth factor receptor-2 and bone morphogenetic protein receptor-2 but with no detectable differences between MP_Ctrl_ and MP_KD-ITSN_ (not shown) and to TGFβ-RII, but in this case, the amounts were 30% less in the MP_KD-ITSN_ compared with the MP_Ctrl_ (Fig. 4E). To get more accurate data regarding the abundance of microparticles and their Alk5 content, we labeled the microparticles with an APC-conjugated antibody against Alk5 and analyzed them by flow cytometry (Fig. 5). Spherotech nano fluorescent size standard beads (0.45 µm, 0.88 µm and 1.35 µm) were used to confirm the size of the microparticles. The microparticle gate was determined using 1.35-µm calibration beads (Fig. 5A, black arrow). For comparison, the red arrow shows counting beads only (3 µm in diameter). The absolute count of MP_Ctrl_ (Fig. 5B) and MP_KD-ITSN_ (Fig. 5C) was measured, setting the stop condition for 1.35-µm beads (upper threshold for microparticle size) at 2000 events. The total number of MP_KD-ITSN_ (129.3×10^3^) shows a ∼1.7-fold increase compared with the total number of MP_Ctrl_ (73.02×10^3^), whereas the number of Alk5-positive MP_KD-ITSN_ (18.8×10^3^) is ∼2.5-fold higher compared with Alk5-positive MP_Ctrl_ (7.52×10^3^; Fig. 5D), consistent with western blotting data. Thus, the components of the vascular system release microparticles comprising Alk5 into the systemic circulation of KD-ITSN mice.

### MP_KD-ITSN_ transfer Alk5 to EC_KD-ITSN_ to restore Erk1/2^MAPK^ pro-survival signaling

**Fig. 6.**
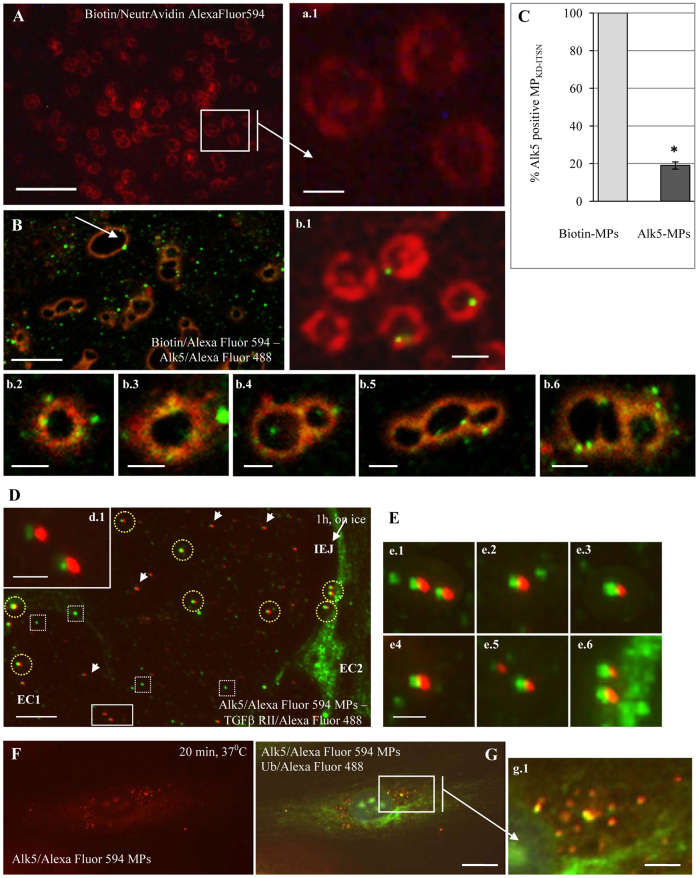
**MP_KD-ITSN_ transfer Alk5 to EC_KD-ITSN_.MP_KD-ITSN_ transfer Alk5 to EC_KD-ITSN_.** (A,a1) Biotin and neutrAvidin–Alexa-Fluor-594 labeling of MP_KD-ITSN_ reveals multiple donut-shaped particles. Double labeling of Alk5 (Alexa Fluor 488) along with biotin and neutrAvidin–Alexa Fluor-594 labeling revealed Alk5 clusters associated with the donut-shaped microparticles (B,b.1–b.6). Light microscopy (b.1) and confocal images (b.2–b.6) illustrate Alk5 immunoreactivity associated with microparticles and the capability of microparticles to fuse with each other (B, arrow; b.4–b.6); the arrow in B indicates a 3–4 µm diameter particle, most likely generated by fusion of individual microparticles due to the relatively long time of processing before 1% paraformaldehyde fixation. (C) Morphometric analyses of Alk5-positive MP_KD-ITSN_. Data are shown as a percentage relative to the control and show the mean±s.e.m.; *P<0.05; *n* = 3. (D) EC_KD-ITSN_ exposed to anti-Alk5–Alexa-Fluor-594 pre-labeled microparticles (MPs) on ice for 1 h were co-stained with anti-TGFβ-RII and an Alexa-Fluor-488-conjugated secondary. Frequent colocalization is suggestive of their heterodimerization and residence on the same microparticle (yellow circles). The endogenous TGFβ-RII is not always associated with microparticle-derived Alk5 (white squares). Even when colocalization of Alk5 and TGFβ–RII is not obvious, Alk5 and TGFβ-RII immunoreactive puncta are found in very close proximity (arrowheads and inset d.1). The arrow in D indicates an interendothelial junction (IEJ). (E,e.1–e.6). Gallery of highly magnified Alk5–TGFβ-RII heterodimers. (F,G) At 37°C, the microparticle-derived Alk5 is detected inside endothelial cells that were counterstained for ubiquitin (Alexa Fluor 488). Scale bars: 10 µm (A,D), 5 µm (B), 1.2 µm (d.1,E), 20 µm (F,G), 1 µm (b.2,b.4,b.5,b.6), 0.5 µm (a.1,b.1,b.3), 4 µm (g.1).

Next, we addressed whether MP_KD-ITSN_ can interact with and transfer Alk5 to endothelial cells, using a microparticle transfer assay and fluorescent imaging. MP_KD-ITSN_, 10 days post siRNA treatment (used throughout the study) were either biotinylated followed by incubation with neutrAvidin conjugated to Alexa Fluor 594 or double labeled with neutrAvidin and Alk5 antibody, followed by streptavidin conjugated to Alexa Fluor 594 and a secondary IgG conjugated to Alexa Fluor 488, as described in Materials and Methods. Biotin–neutrAvidin gives a continuous, donut-shape labeling of microparticles (Fig. 6A,a1). Double biotin and Alk5 antibody labeling revealed Alk5 immunoreactive puncta associated with the donut-shaped particles (Fig. 6B,b.1–b.6); on average, one to four clusters of Alk5 molecules were associated with the donut-shaped, biotin-labeled microparticles. Note also the high propensity of microparticles to fuse to each other (b.4–b.6). The arrow in Fig. 6B points to a large biotin and Alk5-labeled particle (4–5 µm diameter), most likely generated by fusion of two or three individual microparticles. Morphometric analyses indicated that ∼19% of the MP_KD-ITSN_ population is immunoreactive to Alk5 antibody (Fig. 6C), in close agreement with flow cytometry data.

Next, we investigated the ability of MP_KD-ITSN_ to interact (bind and incorporate) and transfer Alk5 to EC_KD-ITSN_. Briefly, MP_KD-ITSN_ were labeled with anti-Alk5 and an Alexa-Fluor-594-conjugated secondary antibody (referred to hereafter as anti-Alk5–Alexa-Fluor-594 pre-labeled MP_KD-ITSN_), using a similar approach to that described above. Biotin–neutrAvidin labeling was omitted to shorten the experimental manipulation of the microparticles to preserve their properties and ability for interaction. Then, EC_KD-ITSN_ at 48 h post siRNA transfection were grown on coverslips and exposed to anti-Alk5–Alexa-Fluor-594 pre-labeled MP_KD-ITSN_ for 1 h on ice to allow binding and, subsequently, transferred to 37°C for 10 min, 20 min and 60 min to allow internalization. Because TGFβ signals through the heteromeric TGFβ-RI–TGFβ-RII receptor complex and because western blotting indicated that the microparticles contained both receptors, the cells were counterstained with a TGFβ-RII antibody followed by an Alexa-Fluor-488-conjugated secondary antibody, to evaluate whether or not the Alk5–TGFβ-RII complex can be detected morphologically on the microparticles interacting with endothelial cells. To this end, EC_KD-ITSN_ exposed to anti-Alk5–Alexa-Fluor-594 pre-labeled MP_KD-ITSN_ for 1 h on ice were subjected to three 10-min washing steps in phosphate buffered saline (PBS), to rule out the possibility of visualizing just the simple physical association of microparticles with the endothelial plasma membrane; then, cells were permeabilized and fixed with methanol at −20°C for 7 min. The permeabilization and fixation step renders endothelial cells unable to internalize the microparticle-derived, pre-labeled Alk5–Alexa-Fluor-594. Fixed and permeabilized endothelial cells were quenched in 1% BSA in PBS and then incubated with TGFβ-RII antibody followed by the appropriate Alexa-Fluor-488-conjugated secondary antibody as described in Materials and Methods. Given this experimental approach, anti-Alk5–Alexa-Fluor-594 labeling indicates only the Alk5 present on the microparticles, whereas the anti-TGFβ-RII–Alexa-Fluor-488 detects both the microparticle-derived and the endogenous receptor. The immunoreactivity for the two receptors is detected frequently colocalizing on the plasma membrane, very suggestive of their heterodimerization and residence on the same microparticle (Fig. 6D, yellow circles; Fig. 6E,e.1–e.6). A lower magnification of the field used to select the image in Fig. 6D is provided in supplementary material Fig. S1. We also detected the endogenous TGFβ-RII not associated with microparticle-derived Alk5 (white squares). Importantly, even if the merged image does not reveal colocalization, the immunoreactivity for the microparticle-derived pre-labeled Alk5 is always in close association with the TGFβ-RII immunoreactivity (Fig. 6D, white arrowheads, d.1). At all time points at 37°C (20 min is shown), the microparticle-derived, Alexa-Fluor-594 pre-labeled Alk5 was detected in the cytosol, consistent with transfer and incorporation of Alk5 from the MP_KD-ITSN_ to EC_KD-ITSN_ (Fig. 6F). Cells were counterstained with ubiquitin antibody (Fig. 6G), followed by the appropriate secondary antibody, for easier identification. Worth mentioning is the significant colocalization between Alk5 and ubiquitin (Fig. 6G, inset g.1), consistent with our hypothesis that, in EC_KD-ITSN_, Alk5 undergoes increased ubiquitylation. To rule out the possibility of non-specific attachment of anti-Alk5 and Alexa-Fluor-594-conjugated IgG aggregates to the MP_KD-ITSN_ and, thus, their endocytic internalization, control experiments were performed using acid-washed microparticles, as described in Materials and Methods. Representative results are shown in supplementary material Fig. S1B.

**Fig. 7.**
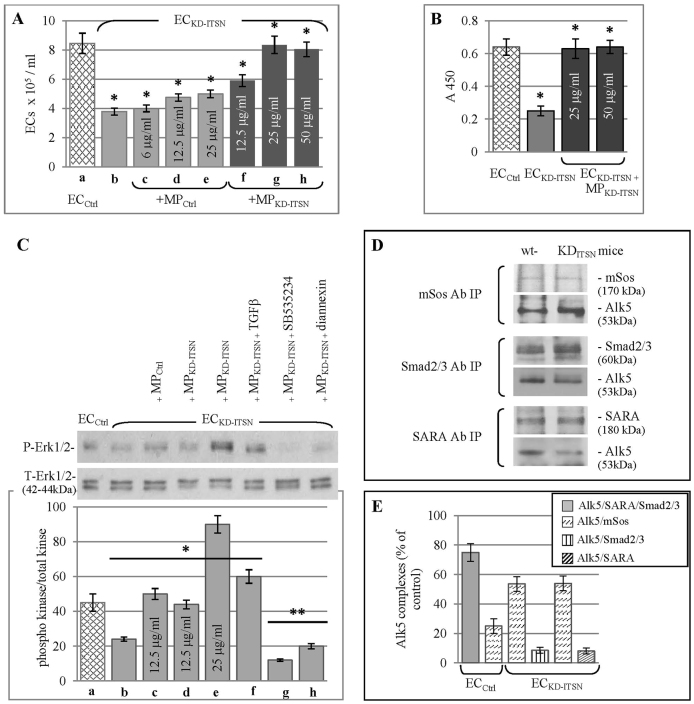
**MP_KD-ITSN_–EC_KD-ITSN_ interaction restores Erk1/2^MAPK^ pro-survival signaling despite ITSN-1s deficiency.MP_KD-ITSN_–EC_KD-ITSN_ interaction restores Erk1/2^MAPK^ pro-survival signaling despite ITSN-1s deficiency.** (A) EC_Ctrl_ (a) and EC_KD-ITSN_ (b–h) were exposed to either MP_Ctrl_ (c–e) or MP_KD-ITSN_ (f–h), as indicated. At 3 days post microparticle exposure, endothelial cells (ECs) were counted and the results expressed as cell number/ml. Data are normalized to day 3 EC_Ctrl_. Values are shown as the mean±s.e.m. *n* = 3; *P<0.05. (B) BrdU incorporation in EC_KD-ITSN_ exposed to MP_KD-ITSN_ compared to EC_KD-ITSN_ without microparticle treatment. Data are normalized to EC_Ctrl_; values are shown as the mean±s.e.m. *n* = 3; *P<0.05. (C) Treatment of EC_KD-ITSN_ with 25 µg/ml MP_KD-ITSN_ increases Erk1/2 phosphorylation (e), by reference to EC_Ctrl_ (a) and EC_KD-ITSN_ (b). MP_Ctrl_ and MP_KD-ITSN_ (12.5 µg/ml) do not significantly activate Erk1/2^MAPK^ (c,d). Exposure of EC_KD-ITSN_ to 10 nM TGFβ for 30 min causes a moderate decrease in Erk1/2 phosphorylation (f), whereas pre-incubation of microparticles with 2 µM/l SB525334 (g) and 2 µM diannexin (h) abolished Erk1/2 activation. Note that frozen microparticles activate Erk1/2 similarly to fresh microparticles. The ratios of phospho (P)-Erk1/2:total (T)-Erk1/2 are shown as the mean±s.e.m.; *n* = 3. *P<0.05; **P<0.01 versus EC_Ctrl_. (D) ITSN-1s deficiency alters the Smad2/3-Erk1/2^MAPK^ signaling balance towards persistent Ras–Erk1/2^MAPK^ activation. EC_KD-ITSN_ lysates were subjected to immunoprecipitation (IP) with mSos, Smad2/3 and SARA antibodies (Ab). The immunoprecipitates were subjected to SDS-PAGE, electrotransferred to nitrocellulose membranes and blotted with antibodies against Alk5, mSos, Smad2/3 and SARA. wt, wild-type. (E) Densitometry of Alk5 levels in Alk5–SARA–Smad2 and Alk5–mSos complexes, considering that EC_KD-ITSN_ show 40% lower Alk5 levels. Data are representative of three experiments performed under identical experimental conditions [500 µg of total protein, 2 µg of each immunoprecipitating antibody, 1∶1000 dilution for secondary antibodies (for Smad2/3 and SARA immunoprecipitaion) and 1∶500 dilution (for mSos immunoprecipitation) and identical ECL exposure time]. The data show the mean±s.e.m.; *P<0.05, versus corresponding EC_Ctrl_ values.

The intercellular microparticle-mediated transfer of Alk5, the downstream signaling molecules of which include Erk1/2^MAPK^ (Derynck and Zhang, 2003), raised the question of whether the survival of EC_KD-ITSN_ *in vivo* might be a consequence of an interaction between microparticles and endothelial cells. Cultured EC_KD-ITSN_, 48 h post siRNA transfection, were exposed to 12.5 µg/ml, 25 µg/ml and 50 µg/ml MP_KD-ITSN_, for 24 h (Fig. 7A,f–h). EC_Ctrl_ (Fig. 7A,a) and EC_KD-ITSN_ (Fig. 7A,b) not exposed to MP_KD-ITSN_ were used for comparison. After 3 days, the cells were counted; despite ITSN-1s deficiency, exposure to 12.5 µg/ml MP_KD-ITSN_ doubled the survival rate of EC_KD-ITSN_ without microparticle exposure (f versus b) and reached 83% of the EC_Ctrl_ number (f versus a); exposure to 25 µg/ml or 50 µg/ml MP_KD-ITSN_ increased more than twofold the survival rate compared to EC_KD-ITSN_ without microparticle exposure (g, h versus b), and reached 98.8% and 95%, respectively, of the EC_Ctrl_ number (g, h versus a). Moreover, exposure of EC_KD-ITSN_ to MP_Ctrl_ (Fig. 7A,c–e) showed only 6%, 26% and 32.5% improvement of survival rate. A ratio of 1∶2 between MP_Ctrl_:MP_KD-ITSN_ was used, to approximate their distribution in the murine systemic circulation.

An enzyme-linked immunosorbent assay (ELISA)-based BrdU cell proliferation assay indicated that EC_KD-ITSN_ exposed for 2 days to 25 µg/ml and 50 µg/ml MP_KD-ITSN_ showed BrdU incorporation similar to that of EC_Ctrl_; however, when compared to EC_KD-ITSN_ without MP_KD-ITSN_ exposure, the BrdU incorporation showed a greater than 2.5-fold increase (Fig. 7B). These data indicate that the intercellular transfer of Alk5 to EC_KD-ITSN_ might rescue EC_KD-ITSN_ from apoptosis. Thus, we next evaluated the effects of the microparticle–EC_KD-ITSN_ interaction on Erk1/2 phosphorylation by western blotting with a phospho-Erk1/2 specific antibody. Exposure of EC_KD-ITSN_ to 12.5 µg/ml or 25 µg/ml MP_KD-ITSN_ resulted in increased Erk1/2 phosphorylation compared with that of EC_KD-ITSN_ without MP_KD-ITSN_ treatment (Fig. 7C,d,e versus b). Erk1/2 phosphorylation reached EC_Ctrl_ values for 12.5 µg/ml MP_KD-ITSN_ (Fig. 7C,d versus a) and was significantly higher when EC_KD-ITSN_ were exposed to 25 µg/ml MP_KD-ITSN_ (Fig. 7C,e versus a). It appears that the interaction between MP_KD-ITSN_ and endothelial cells and a MP_KD-ITSN_ basal threshold are mandatory for Erk1/2 activation and endothelial cell survival following KD- ITSN. EC_KD-ITSN_ without MP_KD-ITSN_ exposure showed 50% lower Erk1/2 phosphorylation compared with that of EC_Ctrl_ (Fig. 7C,b versus a). The blockade of Alk5 by pre-incubation of MP_KD-ITSN_ with 10 µM/l SB525334 (Fig. 7C,g), a selective Alk5 inhibitor, or pre-incubation of MP_KD-ITSN_ with 10 µM diannexin (Fig. 7C,h), an annexin V homodimer known to block the microparticle uptake (del Conde et al., 2005), notably reduced Erk1/2 activation. MP_Ctrl_ did not significantly activate Erk1/2 (Fig. 7C,c). The Alk5 inhibitor affected both the microparticle-derived Alk5 and the endogenous Alk5. EC_KD-ITSN_ not exposed to microparticles (lane b) displayed a low level of Erk1/2 phosphorylation that could be inhibited by 10 µM/l SB525334; the observation is consistent with the idea that the low Erk1/2 activation in EC_KD-ITSN_ is due, at least in part, to the endogenous Alk5 signaling. Exposure of EC_KD-ITSN_ to MP_KD-ITSN_ in the presence of 10 ng/ml TGFβ (Fig. 7C,f), revealed less than a 30% decrease in Erk1/2 phosphorylation compared with EC_KD-ITSN_ exposed to MP_KD-ITSN_ in the absence of TGFβ. However, the degree of Erk1/2 phosphorylation in the presence of TGFβ is still above control levels, consistent with activation of pro-survival signaling and rescue of EC_KD-ITSN_ from apoptotic death caused by ITSN-1s deficiency.

### ITSN-1s deficiency alters the Smad2/3-Erk1/2^MAPK^ signaling balance towards persistent Ras–Erk1/2^MAPK^ activation

ITSN-1s and TGFβ–Alk5 induce Erk1/2^MAPK^ signaling by sharing the same Ras–Raf–MEK cascade (Derynck and Zhang, 2003; Patel et al., 2013; Tong et al., 2000); moreover, ITSN-1s associates with mSos (mammalian Son of sevenless, also known as SOS1) in a protein complex that excludes Grb2 (Tong et al., 2000), raising the possibility that ITSN knockdown might increase mSos availability for Grb2 interaction and, thus, lead to preferential formation of the Alk5–mSos–Grb2 complex and activation of Erk1/2^MAPK^ signaling. Ras–Erk1/2^MAPK^ activation might result in ineffective assembly of Alk5–Smad2–SARA complex and subsequent alteration of the Smad2/3-Erk1/2 signaling balance. To address this possibility, control and KD-ITSN mouse lung lysates were subjected to immunoprecipitation with antibodies against mSos, Smad2/3 and SARA, followed by western blot analyses for Alk5 (Fig. 7D). KD-ITSN mouse lungs showed increased Alk5 association with mSos and decreased association with Smad2/3 and SARA, consistent with non-productive assembly of the Alk5–Smad2/3–SARA complex; no changes in the amounts of mSos, Smad2/3 and SARA immunoprecipitated from EC_Ctrl_ and EC_KD-ITSN_ lysates were detected. Apparently, ITSN-1s deficiency steers Alk5 away from its canonical Smad2/3 signaling and preferentially stimulates the less common Erk1/2^MAPK^ pathway. Based on densitometric analyses of Alk5–Smad2/3, Alk5–Sos and Alk5–SARA interactions and on the finding that Alk5 level in KD-ITSN mouse lung lysates is 40% lower compared to that of controls (Fig. 1A), we determined that in control mouse lungs 75% of Alk5 associates with Smad2/3 and only 25% with mSos. In KD-ITSN mouse lungs, only ∼8% signals through SARA–Smad2/3 and 52% associates with mSos (Fig. 7E). Taken together, the findings are consistent with ineffective assembly of the Alk5–Smad2/3–SARA complex in favor of the Alk5–Sos–Grb2 signaling complex and persistent Ras–Erk1/2^MAPK^ activation with protective effects on lung endothelium.

## DISCUSSION

In the present study, we show that ITSN-1s deficiency in lung endothelial cells and the subsequent endocytic dysfunction result in altered Alk5 intracellular trafficking and enhanced degradation with detrimental consequences for endothelial cell function. Accumulating evidence indicates that perturbation of clathrin- and caveolae-mediated endocytosis functionally upregulates alternative pathways that are either underrepresented or even non-existent under normal conditions (Doherty and McMahon, 2009; Predescu et al., 2012). Recent studies have demonstrated that endocytosis into cav1-dependent tubulovesicular structures, in addition to vesicles, is a common event in mammalian cells (Kirkham et al., 2005; Knezevic et al., 2011; Marbet et al., 2006; Predescu et al., 2012). Moreover, EM studies of the cav1-null mouse revealed the presence of cav1-independent vesicles and vesiculo-vacuolar-like organelles able to mediate transendothelial transport (Predescu et al., 2007b). Our *in vivo* studies indicated that the endocytic deficit generated by modulation of ITSN expression in lung endothelial cells is rescued by upregulation of alternative endocytic pathways and their morphological intermediates (i.e. tubulovesicular and tubular ring-like structures) involved in tracer uptake and transport across endothelium (Knezevic et al., 2011; Predescu et al., 2012). The properties of the cav1-associated tubulovesicular endocytic pathway or clathrin- and cav1-independent pathway and their molecular characteristics are not fully understood; most of the knowledge has been derived mainly from EM studies of the morphology of the endocytic structures in different cell types using different tracers, sensitivity to drugs and their dependence on dynamin (Doherty and McMahon, 2009). Caveolae have been shown to be capable of fusing with the early endosomes in Rab5-dependent processes (Pelkmans et al., 2004). Cav1 is palmitoylated, and it binds cholesterol and fatty acids that might be important in ordering local lipids into invagination-competent compositions (Doherty and McMahon, 2009). Most endocytic pathways, especially cav1-dependent pathways, are sensitive to cholesterol perturbation and are inhibited by the removal of cholesterol (Mayor and Pagano, 2007). Dynamin dependence of the cav1-dependent tubulovesicular structures is unclear; in lung endothelial cells, overexpression of ITSN (Predescu et al., 2003) or of the SH3A domain of ITSN (Knezevic et al., 2011) inhibits the GTPase activity and oligomerization properties of dynamin, resulting in impairment of membrane fission and, thus, formation of membranous tubules, frequently associated with caveolae-like vesicles. Nonetheless, a dynamin pool might escape ITSN-mediated inhibition and, thus, can still mediate the fission of few vesicles and of the tubulovesicular and tubular ring-like structures from the endothelial plasma membrane. In contrast, in EC_KD-ITSN_ characterized by a significant shortage of ITSN scaffold, and thus inefficient dynamin recruitment to the endocytic site, upregulation of the tubulovesicular and tubular ring-like structures is significant. However, other endocytic accessory proteins might partially compensate for dynamin recruitment and membrane invagination and scission, leading to the formation of a few discrete vesicles and release from the plasma membrane of tubulovesicular and tubular ring-like structures.

The endocytic mechanism is vital for many processes including nutrient uptake, membrane recycling, signal transduction and recycling or degradation of cellular receptors (Doherty and McMahon, 2009). In normal endothelial cells, Alk5 is internalized by (1) CCVs, leading to TGFβ-induced Smad2/3 activation, transcriptional responses and recycling to the plasma membrane, and (2) caveolae, which direct Alk5 to the ubiquitin-proteasome and turn off TGFβ signaling (DiGuglielmo et al., 2003). Our studies demonstrate that internalization of Alk5 through the cav1-associated tubulovesicular structures results in enhanced ubiquitylation and degradation, and thereby, decreased Alk5 protein expression. Interestingly, ubiquitylation of some receptor tyrosine kinases promotes association with caveolae and endocytic internalization through a filipin- and nystatin-sensitive, clathrin-independent pathway; in the case of the epidermal growth factor receptor, at high epidermal growth factor concentration, a switch in the endocytic mechanism resulting in receptor ubiquitylation and degradation when endocytosed through caveolae has been reported (Sigismund et al., 2005).

An intriguing observation made during our studies of knocking down ITSN-1s in cultured endothelial cells and mouse lungs relates to the fact that ITSN-1s deficiency in cultured cells triggers mitochondrial apoptosis (Predescu et al., 2007a), whereas, *in vivo*, the peak in endothelial cell apoptosis is followed by survival and alterations of endothelial phenotype towards hyperproliferation and apoptosis resistance (Bardita et al., 2013). The observation raised the possibility that activated or apoptotic cells of KD-ITSN mice generate microparticles that are able to activate pro-survival signaling and modify the endothelial cell phenotype; this is consistent with our results that demonstrate the presence in the systemic circulation of KD-ITSN mice of microparticles bearing the widely expressed Alk5. We have also found that MP_KD-ITSN_ harbor 2.5-fold more Alk5 compared with the MP_Ctrl_. It is of note that microparticles released by cultured EC_KD-ITSN_ do not contain Alk5, and this observation might explain, at least in part, the apoptotic death of cultured endothelial cells. MP_KD-ITSN_ also contain TGFβ-RII, but in smaller amounts compared with MP_Ctrl_. Apparently, Alk5–TGFβ-RII heterodimers are already formed on the microparticles, most likely owing to the TGFβ present in the systemic circulation. Even if our studies do not allow us to draw conclusions on the Alk5 phosphorylation and activation status, the signaling will occur only after Alk5 transfer to EC_KD-ITSN_, which enables Alk5 interaction with downstream partners. Recent studies have shown that receptor endocytosis is not essential for TGFβ signaling (Chen et al., 2009; Lu et al., 2002). Consistent with this, TGFβ–Alk5-mediated Erk1/2 activation can take place on the plasma membrane, without Alk5 endocytic internalization. In addition to Alk5, endothelial cells express Alk1 (also known as SKR3), both involved in TGFβ-induced transcriptional responses, with opposite effects on the activation state of the endothelium; whereas activated Alk5 induces the phosphorylation of Smad2/3, activated Alk1 has been shown to induce the phosphorylation of Smad1/5 (Goumans et al., 2002). TGFβ–Alk1 and TGFβ–Alk5 signaling might be modulated by two accessory TGFβ-R type III receptors – betaglycan (also known as TGFBR3) and endoglin (Lebrin et al., 2005). Because previous reports indicated that TGFβ-RI/Alk5 signaling might be regulated in a ligand-dependent manner by TGFβ co-receptors (Bizet et al., 2012), it is likely these TGFβ co-receptors and accessory proteins account for modulation of Erk1/2 phosphorylation in EC_KD-ITSN_ exposed to microparticles in the presence of TGFβ.

Moreover, MP_KD-ITSN_ readily interact with EC_KD-ITSN_ that contain less Alk5, and they transfer a functional Alk5 receptor, suggesting *in vivo* mechanisms of replenishing EC_KD-ITSN_ with functional Alk5. The functionality of Alk5 is supported by signaling events leading to Erk1/2 kinase phosphorylation and endothelial cell survival; Erk1/2 phosphorylation can be prevented by pre-incubation of MP_KD-ITSN_ with SB-525334, a specific Alk5 inhibitor. The event involves phosphatidyl serine residues of the Alk5-containing microparticles, given that use of diannexin blocks Erk1/2 phosphorylation. Our findings are similar to the recently described transfer of the oncogenic epidermal growth factor receptor present on tumor-derived microparticles to endothelial cells or of the tissue factor present on macrophage-derived microparticles to platelets (Al-Nedawi et al., 2008; del Conde et al., 2005). Circulating microparticles can transfer genetic material and proteins from the donor cells (cells generating the microparticles) to a wide range of target cells, by several mechanisms: internalization and lysosomal processing of microparticles, fusion-mediated transfer of surface receptors, proteins, and lipids, outside-in signaling through ligand–receptor internalization and temporary fusion with the target cell, followed by complete or selective transfer of microparticle content (McVey et al., 2012). Extensive endothelial cell apoptosis caused by KD-ITSN might induce a macrophage phenotype that favors tissue repair and suppression of inflammation (McCubbrey and Curtis, 2013), and as part of this process release microparticles comprising Alk5 into the systemic circulation of the KD-ITSN mice. The ability of apoptotic cells to signal for their non-inflammatory and non-immunogenic removal *in vivo* is crucial for normal tissue homeostasis and for resolution of inflammation (Xiao et al., 2008). Macrophage interaction with apoptotic cells increases the production of TGFβ, which is known to inhibit inflammatory cytokine production through the crosstalk between MAPKs, specifically Erk-dependent inhibition of p38^MAPK^ (Xiao et al., 2002). In addition, experimental and clinical data indicate that platelets are necessarily involved in repair and regeneration of damaged tissues and preservation of organ function (Gawaz and Vogel, 2013). Platelet-derived microparticles constitute the majority of the pool of microparticles circulating in the blood; they express and might transfer functional receptors, stimulate the release of cytokines, activate signaling pathways, promote angiogenesis and participate in tissue regeneration (Varon et al., 2012). Although an increase in TGFβ production by platelets and macrophages as a result of interaction with apoptotic cells has been reported (Dean et al., 2009; Xiao et al., 2002), the release of microparticles enriched in the ubiquitously expressed TGFβ-RI is a novelty of our studies. It is well documented that TGFβ signaling has crucial functional roles in lung development, injury and repair (Warburton et al., 2013). However, it seems that the activated pathways and the end effects of TGFβ signaling are highly dependent on the cellular context and are disease specific. Therefore, it will be of considerable interest to examine whether in human ALI/ARDS patients the number of Alk5-harboring microparticles is increased and whether these particles interact with endothelial cells and impact on the lung vasculature. In most cell types, endothelial cells included, TGFβ signals through TGFβ-RI/Alk5 (Goumans et al., 2002; Lebrin et al., 2005). Although Smad2/3 have been identified as pivotal intracellular effectors of TGFβ–Alk5, there is growing evidence that Ras–Erk1/2^MAPK^ is another major signaling pathway for TGFβ (Derynck and Zhang, 2003). TGFβ induces modest Ras activation consistent with low level Erk1/2 kinase induction (Mulder, 2000). TGFβ-mediated Erk1/2 activation is necessary for TGFβ-induced epithelial-to-mesenchymal transformation (Davies et al., 2005), for regulation of Smad nuclear translocation (Kretzschmar et al., 1999) and for Smad-dependent gene expression (Mucsi et al., 1996). The mechanism by which TGFβ activates Erk1/2 MAP kinases is poorly understood. Our study provides a mechanism whereby MP_KD-ITSN_-derived Alk5 re-wires dysfunctional endothelial cells to activate pro-survival signaling through Erk1/2 kinase and to become hyperproliferative. TGFβ induces Ras–Erk1/2 signaling through phosphorylation of the adaptor protein ShcA (also known as SHC1; Lee et al., 2007), leading to its association with mSos, a Ras GTP/GDP exchange factor, and Grb2 (van der Geer et al., 1996). It appears that ITSN-1s deficiency increases mSos availability for Grb2, favoring the formation of the Alk5–mSos–Grb2 signaling complex. As a result, the assembly of the Alk5–Smad2–SARA signaling complex is unproductive. SARA is a Smad2/3-interacting protein and a control point for Smad2 subcellular localization and TGFβ-dependent transcriptional responses (Tsukazaki et al., 1998). Thus, ITSN deficiency by disturbing the SARA–Smad2 interaction might cause Smad2 subcellular mislocalization. ITSN deficiency also decreases the levels of Smad2/3 phosphorylation (Bardita et al., 2013). Smad2 phosphorylation is required for its association with Smad4, and for the formation and nuclear translocation of the heterotrimeric Smad2/3/4 complex, leading to activation of TGFβ target genes and inhibition of cell proliferation (Goumans et al., 2002; Tsukazaki et al., 1998; Xie et al., 2011). Thus, ITSN deficiency suppresses the Alk5–Smad2/3 pathway, leading to inhibition of the anti-proliferative action of TGFβ. In addition, the TGFβ–Alk5 signaling is switched from the canonical Smad2/3 to the less common Erk1/2^MAPK^ pathway, with protective effects on endothelial cells and lung vasculature. Given that decreased expression of ITSN-1s favors the assembly of Alk5–mSos–Grb2 signaling complexes resulting in downstream Erk1/2 activation, endothelial cells are rescued from apoptotic death caused by ITSN-1s deficiency. The effects induced by MP_KD-ITSN_ on Erk1/2 activation and cell survival are dependent on membrane fusion and Alk5 transfer from microparticles to endothelial cells. Erk1/2 activation is dependent on microparticle number as well, consistent with previous reports that threshold concentrations of biological effectors are important for microparticle-induced physiological effects (Freyssinet, 2003). Although Alk5 transfer might play an important role in rescuing endothelial cells, a possible involvement of other biological effectors that make up microparticles cannot be ruled out. However, this finding might potentially apply also to other cell surface receptors (Predescu et al., 2012), altering their fate, sorting and the functional consequences for proteins involved (Di Fiore and De Camilli, 2001</citref>; Le Roy and Wrana, 2005).

In summary, our studies demonstrate a functional relationship between the intercellular transfer of Alk5 by microparticles and endothelial cell survival and proliferation, and define a novel molecular mechanism for TGFβ–Alk5-dependent Erk1/2^MAPK^ signaling that is significant for the abnormal proliferation of pulmonary endothelial cells.

## MATERIALS AND METHODS

### Endothelial cell culture and siRNA transfection

Human lung microvascular endothelial cell (Lonza, Walkersville, MD) culture and siRNA transfection were performed as described previously (Predescu et al., 2007a). The following siRNA sense sequence was used for knocking down human ITSN-1s: 5′-GGACAUAGUUGUACUGAAAUU-3′ (Dharmacon, Lafayette, CO).

Specific antibodies were against the following proteins (the relevant suppliers are also indicated): Smad-7 (R&D Systems, Minneapolis, MN); Alk5 N-terminal extracellular epitope, Smurf-1, Smad2/3, cav1, SARA, ubiquitin and mSOS (Santa Cruz Biotechnology, Santa Cruz, CA); ITSN-1 (BD Biosciences, San Jose, CA); actin (Sigma-Aldrich, St Louis, MO); Alk5-APC (e-Bioscience, San Diego, CA) and phospho-Erk1/2^MAPK^ (Cell Signaling, Beverly, MA). EM reagents were from EM Sciences (Hatfield, PA). Biotin was from ThermoFisher Scientific (Rockford, IL). All fluorophore-conjugated antibodies and the Prolong Antifade reagent were from Molecular Probes (Eugene, OR). Spherotech nano fluorescent beads were from Spherotech, Inc. (Lake Forest, IL). Flow cytometry reagents were from e-Bioscience (San Diego, CA). SB-525334 and diannexin were from Sigma-Aldrich (St Louis, MO) and human TGFβ1 was from R&D Systems (Minneapolis, MN). Protein-A/G–agarose beads were from Santa Cruz Biotechnology (Santa Cruz, CA).

#### Animals

CD1 male mice, 6–8 weeks old, 20–25 g weight, from Jackson Laboratory (Bar Harbor, ME), kept under standardized housing and feeding conditions were used. The experiments were done under anesthesia, using ketamine (60 mg/kg), acepromazine (2.5 mg/kg) and xylazine (2.5 mg/kg) in 0.1–0.2 ml PBS. </emph>A specific ITSN-1 siRNA sequence (100 µg siRNA/mouse) was delivered by using cationic liposomes, by retro-orbital injection, into mouse lungs as described previously (Bardita et al., 2013; Predescu et al., 2012). The siRNA sense sequence – 5′-GAGAGAGCCAAGCCGGAAAUU-3′ – (Dharmacon, Lafayette, CO) was used for knocking down mouse ITSN-1s. Chronic inhibition of ITSN-1s was achieved by repeated retro-orbital delivery of the siRNA_ITSN_–liposome complexes every 72 h for 24 days as described previously (Predescu et al., 2012). Mice were killed at day 3, day 10, day 15 and day 24; three to four mice per experimental condition [controls (wild-type mice, vehicle- and non-specific siRNA-treated mice) and siRNA_ITSN_-treated mice] were used; all experiments were repeated at least three times. No mouse mortality was recorded during the 24 days of the study. All experiments were approved and performed in accordance with the guidelines of Rush University Institutional Animal Care and Use Committee.

### Isolation of microparticles

Blood of fully anesthetized wild-type and KD_ITSN_ mice was drawn by cardiac puncture and using 3.8% sodium citrate as an anticoagulant. Platelet-free plasma was centrifuged at 80,000 ***g*** for 2 h at 4°C to obtain the microparticle pellets; microparticles were either lysed or used intact for morphological approaches. All morphological approaches were performed with freshly isolated microparticles.

### Fluorescent labeling of microparticles and immunofluorescent staining

Microparticles were incubated with 1 mg/ml biotin in PBS containing 0.1 M CaCl_2_ and 0.1 M MgCl_2_ for 20 min on ice, followed by incubation with neutrAvidin–Alexa-Fluor-594 diluted in 0.1% BSA in PBS for 1 h. The unbound biotin and neutrAvidin–Alexa-Fluor-594 were removed by three successive washings in PBS followed by centrifugation (Beckman centrifuge,TLA-55 rotor) at 80,000 ***g*** for 1 h at 4°C. For double labeling with biotin and Alk5 antibody, microparticles were sequentially incubated with: (1) Alk5 goat primary antibody diluted in 0.1% BSA in PBS, overnight at 4°C, followed by (2) biotin, as above and (3) a mixture of neutrAvidin–Alexa-Fluor-594 and anti-goat-IgG conjugated to Alexa Fluor 488, for 1 h at room temperature. A blocking step using 1% BSA in PBS preceded incubation with the Alk5 antibody. Successive washings in 0.1% BSA in PBS followed by centrifugation were used to remove excess biotin or antibodies. Final pellets were resuspended in PBS and fixed in 1% paraformaldehyde, and aliquots were mounted on glass slides with Prolong Antifade reagent. Isotype-matched IgG was used as a control. Microparticles were examined and photographed using a Zeiss AxioImager M1 microscope or Zeiss Laser Scanning Microscope LSM 700.

Immunofluorescent staining of endothelial cells grown on coverslips was performed as described previously (Predescu et al., 2005). Incubation of endothelial cells with isotype-matched IgGs or omission of the primary antibody were used as controls for antibody specificity. Each set of experiments was performed in triplicate.

### Morphometric analysis

The degree of cav1 and Alk5 colocalization was determined by counting the cav1-positive large endocytic structures in 50 endothelial cells per coverslip, in three different experiments performed in triplicate. All images used for quantification of the degree of colocalization were acquired using identical parameters per experiment.

### Microparticle–endothelial cell interaction

Endothelial cells were grown on coverslips and exposed for 1 h on ice to 12.5 µg/ml MP_KD-ITSN_ pre-labeled with an anti-Alk5 antibody and an Alexa-Fluor-594-conjugated secondary antibody, to allow binding of the microparticles to the endothelial plasma membrane; then, cells were transferred to 37°C for 15 min and 30 min, to allow internalization. Cells were washed, fixed in methanol for 7 min at −20°C, quenched in 1% BSA/PBS for 1 h at room temperature and counterstained by incubation with TGFβ-RII and ubiquitin antibodies, followed by their specific secondary antibodies, as above. Cells were examined and photographed using a Zeiss AxioImager M1 microscope.

Control experiments to rule out the endocytic internalization by EC_KD-ITSN_ of non-specifically attached anti-Alk5–Alexa-Fluor-594 IgG aggregates to the MP_KD-ITSN_ were performed. Briefly, anti-Alk5–Alexa-Fluor-594 pre-labeled MP_KD-ITSN_ were resuspended in ice-cold acid wash buffer (DMEM/10 mM HEPES pH 5.0, 10 mM MES, 120 mM NaCl, 0.5 mM MgCl_2_ and 0.9 mM CaCl_2_) for 30 min as described previously (Koenig et al., 1997; Smalley et al., 2001). At this pH, the MP_KD-ITSN_ are not stripped of their pre-labeled Alk5. The acid-wash buffer was removed by ultra-centrifugation and the MP_KD-ITSN_ pellet was resuspended in DMEM containing 0.1% BSA. EC_KD-ITSN_ grown on coverslips were exposed to a mixture of 12.5 µg/ml anti-Alk5–Alexa-Fluor-594 pre-labeled MP_KD-ITSN_ and unlabeled Alk5 antibody (dilution 1∶ 1000; for 1 h on ice, to allow binding of anti-Alk5–Alexa-Fluor-594 pre-labeled MP_KD-ITSN_ to the endothelial plasma membrane and to block the endogenous Alk5 receptor, respectively. The cells were transferred to 37°C, for 20 min to allow internalization as above.

### Flow cytometry

Microparticles were isolated from wild-type and KD-ITSN mice and labeled with an APC-conjugated anti-Alk5 antibody diluted in flow cytometry staining buffer, according to the manufacturer's indications. Samples were incubated for 1 h at 4°C in the dark, and then centrifuged for 1 h at 80,000 ***g***. Pellets were resuspended in 1 ml of staining buffer and centrifuged, with this procedure being repeated three times to remove excess antibody. The final pellet was resuspended in 50 µl and analyzed in a LSR Fortessa flow cytometer with Diva software. Control experiments included incubation with isotype control mouse IgG. Microparticle gating was accomplished by preliminary standardization experiments using Spherotech nano fluorescent size standard beads (0.45 µm–1.35 µm). Data are presented as dot plots and the results of data analysis are presented as the mean percentage of total gated events (at least 10,000 events/sample) ±s.e.m.

### Activation of Erk1/2^MAPK^

Western blotting using anti-phospho-Erk1/2^MAPK^ antibody as described previously (Bardita et al., 2013) was performed with lysates of EC_Ctrl_ and EC_KD-ITSN_-exposed microparticles. Cells were starved for 2 h prior to microparticle exposure. For some experiments, microparticles were pre-incubated with 2 µM/l SB-525334 (Laping et al., 2007), with 2 µM diannexin (Al-Nedawi et al., 2008) or with human TGFβ1 (10 ng/ml) for 30 min, added simultaneously with microparticles to the endothelial cell medium (Kavsak et al., 2000).

### Immunoprecipitation and western blot analyses

All these procedures were performed as described previously (Predescu et al., 2001; Predescu et al., 2003). KD_ITSN_ mice were killed at 3 days, 6 days, 10 days, 15 days and 24 days post-siRNA_ITSN_ initiation; lungs were excised and homogenized in 150 mM NaCl, 50 mM Tris-HCl pH 8.0 and protease inhibitors; lysates were prepared by adding NP-40 to a final concentration of 1.0%, and samples were incubated for 2 h at 4°C, followed by centrifugation (Beckman ultracentrifuge, TLA-55 rotor) for 45 min at 4°C and 45,000 rpm. Protein concentration was determined by the microBCA method. The microparticle lysates were prepared as above. The mouse lung lysates (70 µg total protein/lane), endothelial cell or microparticle lysates (50 µg protein/lane) were analyzed by SDS-PAGE and electrotransferred to nitrocellulose membranes, which were probed with antibodies against the following proteins: Alk5 (1∶1000), ITSN-1 (1∶500), actin (1∶2000), ubiquitin (1∶200), phospho-Erk1/2 and total Erk1/2 (1∶1000), TGFβRII (1∶500), Smad7 (1∶1000) and Smurf1 (1∶1000). Bound antibodies were visualized by using enhanced chemiluminescence. For immunoprecipitation, 500 µg of total protein from lung lysates or endothelial cells was pre-cleared and then incubated with 2 µg each of antibodies against Smad2/3, Alk5, mSos and SARA, followed by Protein-A/G–agarose beads. The immunoprecipitates were analyzed by 5–20% SDS-PAGE. The gels were transferred to nitrocellulose membranes followed by western blotting, as above. For detection of Alk5 ubiquitylation by western blotting, 5 mM N-ethylmaleimide was added to the immunoprecipitation buffer to prevent the cleavage of polyubiquitin chains (Mata-Greenwood et al., 2013).

### Negative staining and pre-embedding immuno-EM

Microparticles were fixed in 2.5% glutaraldehyde for 30 min at room temperature, absorbed onto formvar-coated nickel grids recently exposed to glow discharge and negatively stained as described previously (Predescu et al., 2001). EM grids were analyzed in a JEOL JEM-2000FX TEM. For Alk5 pre-embedding immuno-EM, thick cryostat sections of polyvinylpyrrolidone-fixed tissue were incubated with anti-Alk5 antibody followed by goat anti-rat-IgG conjugated to 8-nm gold and processed by standard EM procedure as described previously (Predescu et al., 1996).

### Statistical analysis

All findings were confirmed in three to five different experiments and data are expressed as the mean±s.e.m. Stimulated samples were compared to controls by using unpaired Student's *t*-tests. Differences with values of *P*<0.05 were considered to be statistically significant.

## Supplementary Material

Supplementary Material

## References

[b01] Al-Nedawi K, Meehan B, Micallef J, Lhotak V, May L, Guha A, Rak J (2008). Intercellular transfer of the oncogenic receptor EGFRvIII by microvesicles derived from tumour cells.. Nat. Cell Biol..

[b02] Bardita C, Predescu D, Justice MJ, Petrache I, Predescu S (2013). In vivo knockdown of intersectin-1s alters endothelial cell phenotype and causes microvascular remodeling in the mouse lungs.. Apoptosis.

[b03] Bizet AA, Tran-Khanh N, Saksena A, Liu K, Buschmann MD, Philip A (2012). CD109-mediated degradation of TGF-β receptors and inhibition of TGF-β responses involve regulation of SMAD7 and Smurf2 localization and function.. J. Cell. Biochem..

[b04] Chen CL, Hou WH, Liu IH, Hsiao G, Huang SS, Huang JS (2009). Inhibitors of clathrin-dependent endocytosis enhance TGFbeta signaling and responses.. J. Cell Sci..

[b05] D'Alessio FR, Tsushima K, Aggarwal NR, West EE, Willett MH, Britos MF, Pipeling MR, Brower RG, Tuder RM, McDyer JF (2009). CD4+CD25+Foxp3+ Tregs resolve experimental lung injury in mice and are present in humans with acute lung injury.. J. Clin. Invest..

[b06] Davies M, Robinson M, Smith E, Huntley S, Prime S, Paterson I (2005). Induction of an epithelial to mesenchymal transition in human immortal and malignant keratinocytes by TGF-beta1 involves MAPK, Smad and AP-1 signalling pathways.. J. Cell. Biochem..

[b07] Dean WL, Lee MJ, Cummins TD, Schultz DJ, Powell DW (2009). Proteomic and functional characterisation of platelet microparticle size classes.. Thromb. Haemost..

[b08] del Conde I, Shrimpton CN, Thiagarajan P, López JA (2005). Tissue-factor-bearing microvesicles arise from lipid rafts and fuse with activated platelets to initiate coagulation.. Blood.

[b09] Derynck R, Zhang YE (2003). Smad-dependent and Smad-independent pathways in TGF-beta family signalling.. Nature.

[b10] Di Fiore PP, De Camilli P (2001). Endocytosis and signaling. an inseparable partnership.. Cell.

[b11] Di Guglielmo GM, Le Roy C, Goodfellow AF, Wrana JL (2003). Distinct endocytic pathways regulate TGF-beta receptor signalling and turnover.. Nat. Cell Biol..

[b12] Doherty GJ, McMahon HT (2009). Mechanisms of endocytosis.. Annu. Rev. Biochem..

[b13] Freyssinet JM (2003). Cellular microparticles: what are they bad or good for?. J. Thromb. Haemost..

[b14] Gawaz M, Vogel S (2013). Platelets in tissue repair: control of apoptosis and interactions with regenerative cells.. Blood.

[b15] Goumans MJ, Valdimarsdottir G, Itoh S, Rosendahl A, Sideras P, ten Dijke P (2002). Balancing the activation state of the endothelium via two distinct TGF-beta type I receptors.. EMBO J..

[b16] Henson PM, Tuder RM (2008). Apoptosis in the lung: induction, clearance and detection.. Am. J. Physiol..

[b17] Itoh S, ten Dijke P (2007). Negative regulation of TGF-beta receptor/Smad signal transduction.. Curr. Opin. Cell Biol..

[b18] Kavsak P, Rasmussen RK, Causing CG, Bonni S, Zhu H, Thomsen GH, Wrana JL (2000). Smad7 binds to Smurf2 to form an E3 ubiquitin ligase that targets the TGF beta receptor for degradation.. Mol. Cell.

[b19] Kirkham M, Fujita A, Chadda R, Nixon SJ, Kurzchalia TV, Sharma DK, Pagano RE, Hancock JF, Mayor S, Parton RG (2005). Ultrastructural identification of uncoated caveolin-independent early endocytic vehicles.. J. Cell Biol..

[b20] Knezevic I, Predescu D, Bardita C, Wang M, Sharma T, Keith B, Neamu R, Malik AB, Predescu S (2011). Regulation of dynamin-2 assembly-disassembly and function through the SH3A domain of intersectin-1s.. J. Cell. Mol. Med..

[b21] Koenig JA, Edwardson JM, Humphrey PP (1997). Somatostatin receptors in Neuro2A neuroblastoma cells: operational characteristics.. Br. J. Pharmacol..

[b22] Kranenburg AR, De Boer WI, Van Krieken JH, Mooi WJ, Walters JE, Saxena PR, Sterk PJ, Sharma HS (2002). Enhanced expression of fibroblast growth factors and receptor FGFR-1 during vascular remodeling in chronic obstructive pulmonary disease.. Am. J. Respir. Cell Mol. Biol..

[b23] Kretzschmar M, Doody J, Timokhina I, Massagué J (1999). A mechanism of repression of TGFbeta/ Smad signaling by oncogenic Ras.. Genes Dev..

[b24] Laping NJ, Everitt JI, Frazier KS, Burgert M, Portis MJ, Cadacio C, Gold LI, Walker CL (2007). Tumor-specific efficacy of transforming growth factor-beta RI inhibition in Eker rats.. Clin. Cancer Res..

[b25] Le A, Damico R, Damarla M, Boueiz A, Pae HH, Skirball J, Hasan E, Peng X, Chesley A, Crow MT (2008). Alveolar cell apoptosis is dependent on p38 MAP kinase-mediated activation of xanthine oxidoreductase in ventilator-induced lung injury.. J. Appl. Physiol..

[b26] Le Roy C, Wrana JL (2005). Clathrin- and non-clathrin-mediated endocytic regulation of cell signalling.. Nat. Rev. Mol. Cell Biol..

[b27] Lebrin F, Deckers M, Bertolino P, Ten Dijke P (2005). TGF-beta receptor function in the endothelium.. Cardiovasc. Res..

[b28] Lee MK, Pardoux C, Hall MC, Lee PS, Warburton D, Qing J, Smith SM, Derynck R (2007). TGF-beta activates Erk MAP kinase signalling through direct phosphorylation of ShcA.. EMBO J..

[b29] Lu Z, Murray JT, Luo W, Li H, Wu X, Xu H, Backer JM, Chen YG (2002). Transforming growth factor beta activates Smad2 in the absence of receptor endocytosis.. J. Biol. Chem..

[b30] Marbet P, Rahner C, Stieger B, Landmann L (2006). Quantitative microscopy reveals 3D organization and kinetics of endocytosis in rat hepatocytes.. Microsc. Res. Tech..

[b31] Mata-Greenwood E, Stewart JM, Steinhorn RH, Pearce WJ (2013). Role of BCL2-associated athanogene 1 in differential sensitivity of human endothelial cells to glucocorticoids.. Arterioscler. Thromb. Vasc. Biol..

[b32] Mause SF, Weber C (2010). Microparticles: protagonists of a novel communication network for intercellular information exchange.. Circ. Res..

[b33] Mayor S, Pagano RE (2007). Pathways of clathrin-independent endocytosis.. Nat. Rev. Mol. Cell Biol..

[b34] McCubbrey AL, Curtis JL (2013). Efferocytosis and lung disease.. Chest.

[b35] McVey M, Tabuchi A, Kuebler WM (2012). Microparticles and acute lung injury.. Am. J. Physiol..

[b36] Morrell NW, Yang X, Upton PD, Jourdan KB, Morgan N, Sheares KK, Trembath RC (2001). Altered growth responses of pulmonary artery smooth muscle cells from patients with primary pulmonary hypertension to transforming growth factor-beta(1) and bone morphogenetic proteins.. Circulation.

[b37] Mucsi I, Skorecki KL, Goldberg HJ (1996). Extracellular signal-regulated kinase and the small GTP-binding protein, Rac, contribute to the effects of transforming growth factor-beta1 on gene expression.. J. Biol. Chem..

[b38] Mukherjee S, Tessema M, Wandinger-Ness A (2006). Vesicular trafficking of tyrosine kinase receptors and associated proteins in the regulation of signaling and vascular function.. Circ. Res..

[b39] Mulder KM (2000). Role of Ras and Mapks in TGFbeta signaling.. Cytokine Growth Factor Rev..

[b40] Murakami K, Mathew R, Huang J, Farahani R, Peng H, Olson SC, Etlinger JD (2010). Smurf1 ubiquitin ligase causes downregulation of BMP receptors and is induced in monocrotaline and hypoxia models of pulmonary arterial hypertension.. Exp. Biol. Med. (Maywood).

[b41] Patel M, Predescu D, Tandon R, Bardita C, Pogoriler J, Bhorade S, Wang M, Comhair S, Hemnes AR, Chen J (2013). A novel p38 mitogen-activated protein kinase/Elk-1 transcription factor-dependent molecular mechanism underlying abnormal endothelial cell proliferation in plexogenic pulmonary arterial hypertension.. J. Biol. Chem..

[b42] Pelkmans L, Bürli T, Zerial M, Helenius A (2004). Caveolin-stabilized membrane domains as multifunctional transport and sorting devices in endocytic membrane traffic.. Cell.

[b43] Predescu D, Ihida K, Predescu S, Palade GE (1996). The vascular distribution of the platelet-activating factor receptor.. Eur. J. Cell Biol..

[b44] Predescu SA, Predescu DN, Palade GE (2001). Endothelial transcytotic machinery involves supramolecular protein-lipid complexes.. Mol. Biol. Cell.

[b45] Predescu SA, Predescu DN, Timblin BK, Stan RV, Malik AB (2003). Intersectin regulates fission and internalization of caveolae in endothelial cells.. Mol. Biol. Cell.

[b46] Predescu SA, Predescu DN, Shimizu K, Klein IK, Malik AB (2005). Cholesterol-dependent syntaxin-4 and SNAP-23 clustering regulates caveolar fusion with the endothelial plasma membrane.. J. Biol. Chem..

[b47] Predescu SA, Predescu DN, Knezevic I, Klein IK, Malik AB (2007a). Intersectin-1s regulates the mitochondrial apoptotic pathway in endothelial cells.. J. Biol. Chem..

[b48] Predescu SA, Predescu DN, Malik AB (2007b). Molecular determinants of endothelial transcytosis and their role in endothelial permeability.. Am. J. Physiol..

[b49] Predescu DN, Neamu R, Bardita C, Wang M, Predescu SA (2012). Impaired caveolae function and upregulation of alternative endocytic pathways induced by experimental modulation of intersectin-1s expression in mouse lung endothelium.. Biochem. Res. Int..

[b50] Predescu DN, Bardita C, Tandon R, Predescu SA (2013). Intersectin-1s: an important regulator of cellular and molecular pathways in lung injury.. Pulmonary Circulation.

[b51] Ranieri VM, Rubenfeld GD, Thompson BT, Ferguson ND, Caldwell E, Fan E, Camporota L, Slutsky AS, ARDS Definition Task Force (2012). Acute respiratory distress syndrome: the Berlin Definition.. JAMA.

[b52] Schmidt EP, Tuder RM (2010). Role of apoptosis in amplifying inflammatory responses in lung diseases.. J. Cell Death..

[b53] Sehgal PB, Mukhopadhyay S (2007). Dysfunctional intracellular trafficking in the pathobiology of pulmonary arterial hypertension.. Am. J. Respir. Cell Mol. Biol..

[b54] Sigismund S, Woelk T, Puri C, Maspero E, Tacchetti C, Transidico P, Di Fiore PP, Polo S (2005). Clathrin-independent endocytosis of ubiquitinated cargos.. Proc. Natl. Acad. Sci. USA.

[b55] Smalley KS, Koenig JA, Feniuk W, Humphrey PP (2001). Ligand internalization and recycling by human recombinant somatostatin type 4 (h sst(4)) receptors expressed in CHO-K1 cells.. Br. J. Pharmacol..

[b56] Sorkin A, von Zastrow M (2009). Endocytosis and signalling: intertwining molecular networks.. Nat. Rev. Mol. Cell Biol..

[b57] Tong XK, Hussain NK, de Heuvel E, Kurakin A, Abi-Jaoude E, Quinn CC, Olson MF, Marais R, Baranes D, Kay BK (2000). The endocytic protein intersectin is a major binding partner for the Ras exchange factor mSos1 in rat brain.. EMBO J..

[b58] Tsukazaki T, Chiang TA, Davison AF, Attisano L, Wrana JL (1998). SARA, a FYVE domain protein that recruits Smad2 to the TGFbeta receptor.. Cell.

[b59] van der Geer P, Wiley S, Gish GD, Pawson T (1996). The Shc adaptor protein is highly phosphorylated at conserved, twin tyrosine residues (Y239/240) that mediate protein-protein interactions.. Curr. Biol..

[b60] Varon D, Hayon Y, Dashevsky O, Shai E (2012). Involvement of platelet derived microparticles in tumor metastasis and tissue regeneration.. Thromb. Res..

[b61] Voelkel NF, Cool CD (2003). Pulmonary vascular involvement in chronic obstructive pulmonary disease.. Eur. Respir. J. Suppl..

[b62] Warburton D, Shi W, Xu B (2013). TGF-β-Smad3 signaling in emphysema and pulmonary fibrosis: an epigenetic aberration of normal development?. Am. J. Physiol..

[b63] Wiener-Kronish JP, Albertine KH, Matthay MA (1991). Differential responses of the endothelial and epithelial barriers of the lung in sheep to Escherichia coli endotoxin.. J. Clin. Invest..

[b64] Xiao YQ, Malcolm K, Worthen GS, Gardai S, Schiemann WP, Fadok VA, Bratton DL, Henson PM (2002). Cross-talk between ERK and p38 MAPK mediates selective suppression of pro-inflammatory cytokines by transforming growth factor-beta.. J. Biol. Chem..

[b65] Xiao YQ, Freire-de-Lima CG, Schiemann WP, Bratton DL, Vandivier RW, Henson PM (2008). Transcriptional and translational regulation of TGF-beta production in response to apoptotic cells.. J. Immunol..

[b66] Xie L, Vo-Ransdell C, Abel B, Willoughby C, Jang S, Sowa G (2011). Caveolin-2 is a negative regulator of anti-proliferative function and signaling of transforming growth factor-β in endothelial cells.. Am. J. Physiol..

