# Supplementary material for: Endocytic deficiency induced by ITSN-1s knockdown alters the Smad2/3-Erk1/2 signaling balance downstream of Alk5
Source: J Cell Sci. 2015 Apr 15;128(8):1528–41. doi: 10.1242/jcs.163030 (PMC4406123; doi:10.1242/jcs.163030)
Supplement: Supplementary Material [file supp_128_8_1528__index.html]

Supplementary Material 

# Endocytic deficiency induced by ITSN‐1s knockdown alters the Smad2/3‐Erk1/2 signaling balance downstream of Alk5

## JCS163030 Supplementary Material

**Files in this Data Supplement:**

- **Supplementary Material**
