# Supplementary material for: Endocytic deficiency induced by ITSN-1s knockdown alters the Smad2/3-Erk1/2 signaling balance downstream of Alk5
Source: J Cell Sci. 2015 Apr 15;128(8):1528–41. doi: 10.1242/jcs.163030 (PMC4406123; doi:10.1242/jcs.163030)
Supplement: Supplementary Material [file supp_128.8.1528_JCS163030.pdf]

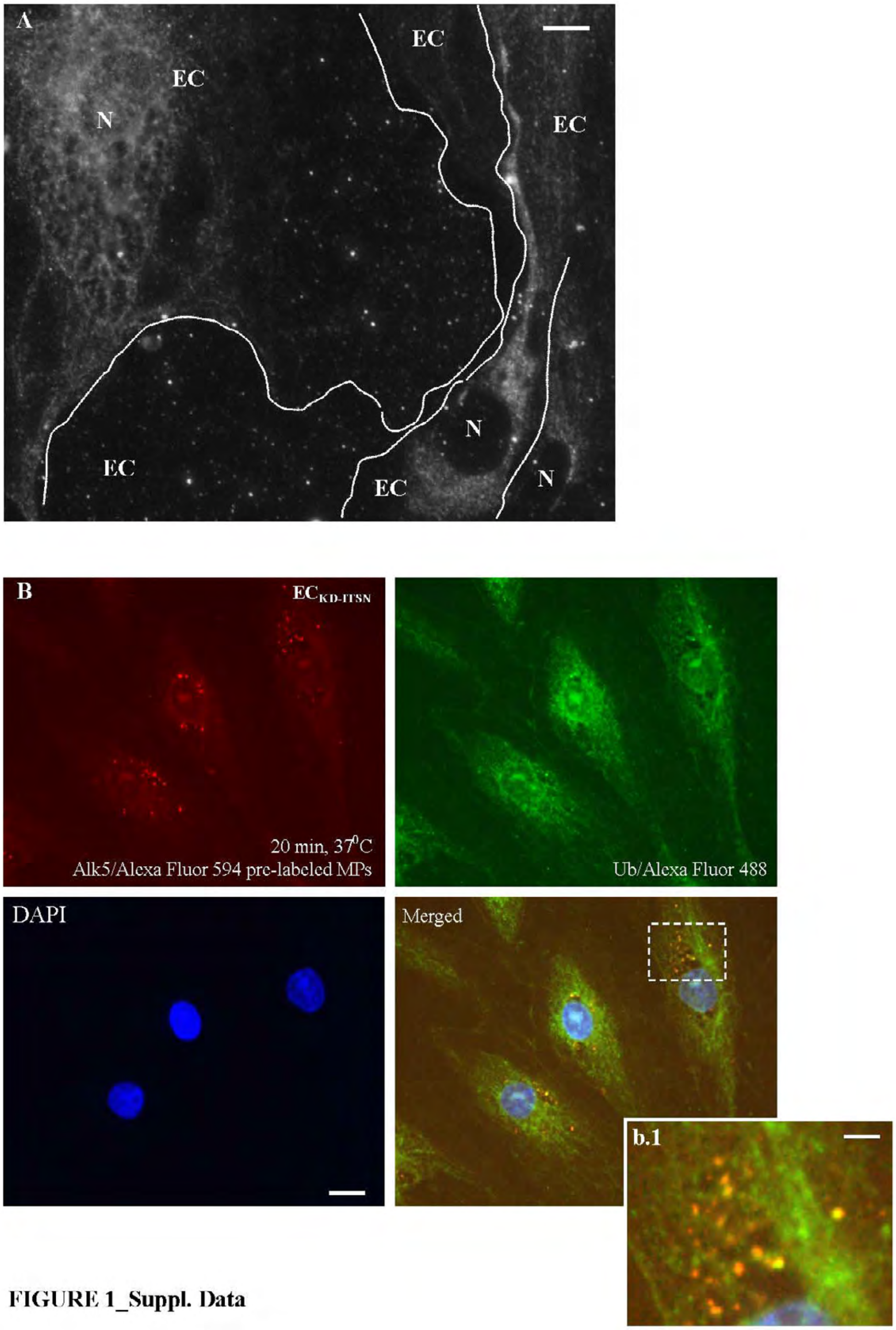


**Figure S1**

**A.** $EC_{KD\text{-}ITSN}$ exposed to Alk5/Alexa Fluor 594 pre-labeled MPs, on ice for 1h, were co-stained with anti-TGFβ-RII Ab/Alexa Fluor 488-conjugated reporter (green channel is shown). EC-endothelial cell; N – nucleus. **B.** Representative micrographs of $EC_{KD\text{-}ITSN}$ exposed to acid-washed Alk5/Alexa Fluor 594 pre-labeled $MP_{KD\text{-}ITSN}$ and unlabelled Alk5 rabbit Ab, on ice for 1h, followed by 20 min at 37$^0$C incubation. Cells were co-stained with anti Ub mouse Ab/Alexa Fluor 488-conjugated reporter. Bars: 10 mm (A); 20 mm (B), 5 mm (b.1).
